# Genetic testing in steroid-resistant nephrotic syndrome: why, who, when and how?

**DOI:** 10.1007/s00467-017-3838-6

**Published:** 2017-11-27

**Authors:** Rebecca Preston, Helen M. Stuart, Rachel Lennon

**Affiliations:** 1 Division of Cell Matrix Biology, Wellcome Trust Centre for Cell-Matrix Research, School of Biological Sciences, Faculty of Biology Medicine and Health, University of Manchester, Manchester, UK; 2 Division of Evolution and Genomic Sciences, School of Biological Sciences, Faculty of Biology, Medicine and Health, The University of Manchester, Manchester, UK; 3 Manchester Centre for Genomic Medicine, St. Mary’s Hospital, Central Manchester Foundation NHS Trust, Manchester Academic Health Science Centre (MAHSC), Manchester, UK; 4 Department of Paediatric Nephrology, Royal Manchester Childrens’ Hospital, Central Manchester University Hospitals NHS Foundation Trust, Manchester Academic Health Science Centre, Manchester, UK

**Keywords:** Steroid-resistant nephrotic syndrome, Focal segmental glomerulosclerosis, Monogenic, Mutational screening, Genetic testing

## Abstract

Steroid-resistant nephrotic syndrome (SRNS) is a common cause of chronic kidney disease in childhood and has a significant risk of rapid progression to end-stage renal disease. The identification of over 50 monogenic causes of SRNS has revealed dysfunction in podocyte-associated proteins in the pathogenesis of proteinuria, highlighting their essential role in glomerular function. Recent technological advances in high-throughput sequencing have enabled indication-driven genetic panel testing for patients with SRNS. The availability of genetic testing, combined with the significant phenotypic variability of monogenic SRNS, poses unique challenges for clinicians when directing genetic testing. This highlights the need for clear clinical guidelines that provide a systematic approach for mutational screening in SRNS. The likelihood of identifying a causative mutation is inversely related to age at disease onset and is increased with a positive family history or the presence of extra-renal manifestations. An unequivocal molecular diagnosis could allow for a personalised treatment approach with weaning of immunosuppressive therapy, avoidance of renal biopsy and provision of accurate, well-informed genetic counselling. Identification of novel causative mutations will continue to unravel the pathogenic mechanisms of glomerular disease and provide new insights into podocyte biology and glomerular function.

## Introduction

Nephrotic syndrome (NS) comprises a heterogeneous group of disorders characterised by hypoalbuminaemia, oedema and hyperlipidaemia. This primarily reflects dysfunction of the normally size- and charge-selective glomerular filtration barrier (GFB), with resultant loss of protein into the urine. NS is the most common glomerular disease of childhood, with an estimated incidence of approximately 1–2 per 100,000 children [1, 2], accounting for approximately 10% of early-onset chronic kidney disease [3]. Classification is based on the response to treatment with glucocorticoids (Gc) as either steroid-sensitive (where Gc induces remission) or steroid-resistant NS (SRNS). Approximately 80% of paediatric NS cases respond to Gc, with the remaining 20% being steroid-resistant [4]. SRNS may be further characterised by renal histology, with the majority of cases showing focal segmental glomerulosclerosis (FSGS) [5] and, to a lesser extent, minimal change disease (MCD) or diffuse mesangial sclerosis (DMS). Furthermore, SRNS may occur as an isolated kidney disease or, less frequently, as a syndromic disorder associated with extra-renal manifestations. There is significant heterogeneity in the onset and clinical course of SRNS, and neither the clinical features nor the histological pattern predicts therapy response. However, SRNS is more likely to show resistance to a range of immunosuppressive agents [6] and progress to end-stage renal disease (ESRD) at a faster rate [4, 7].

The exponential discovery of genes implicated in SRNS has helped to build understanding about the molecular mechanisms of glomerular filtration. Mutations in genes encoding podocyte-associated proteins have been implicated in about 30% of SRNS cases in children [8–10] (Table 1), and identification of these monogenic defects has provided fundamental insights into the pathogenesis of SRNS. Importantly, monogenic SRNS exhibits significant clinical and histological heterogeneity, even with identical causative mutations, and is initially indistinguishable from idiopathic NS. However, children with monogenic SRNS experience higher rates of resistance to immunosuppression and lower rates of disease reoccurrence after renal transplantation [6, 63].

**Table 1:** Monogenic causes of steroid-resistant nephrotic syndrome identified to date, including details of associated clinical phenotype, most frequently observed renal histological lesion and likely mode of inheritance

Gene	Protein	Phenotype	Mode of inheritance	Histology	Reference
Slit diaphragm-associated proteins					
*NPHS1*	Nephrin	CNS (Finnish type), SRNS (early onset)	AR	PTRD, PMS, FSGS, MCD	[11]
*NPHS2*	Podocin	CNS, SRNS (early and late onset)	AR	FSGS, MCD	[12]
*PLCE1*	Phospholipase C epsilon 1	CNS, SRNS (early onset)	AR	DMS, FSGS	[13]
*CD2AP*	CD2-associated protein	SRNS	AD, AR	FSGS	[14]
*TRPC6*	Transient receptor potential channel C6	SRNS (late onset)	AD	FSGS	[15]
*CRB2*	Crumbs family member 2	SRNS	AR	FSGS	[16]
*FAT1*	FAT atypical cadherin 1	FSGS, neurological involvement	AR	Variable	[17]
Nuclear proteins and transcription factors					
*WT1*	Wilms’ tumour protein 1	Denys Drash, Frasier, isolated SRNS +/− ambiguous genitalia	AD, AR	FSGS, DMS	[18, 19]
*LMX1B*	LIM homeobox transcription factor 1β	Nail-patella syndrome, isolated SRNS	AD	FSGS	[20]
*SMARCL1*	SMARCA-like protein	Schimke immuno-osseous dysplasia	AR	FSGS	[21]
*NUP93*	Nuclear pore complex protein 93	SRNS	AR	FSGS	[22]
*NUP107*	Nuclear pore complex protein 107	SRNS (early onset)	AR	FSGS	[23]
*NUP205*	Nuclear pore complex protein 205	SRNS	AR	FSGS	[22]
*XPO5*	Exportin 5	SRNS	AR	FSGS	[22]
*E2F3*	E2F transcription factor	FSGS, mental retardation (gene deletion)	AD	FSGS	[24]
*NXF5*	Nuclear RNA export Factor 5	FSGS, co-segregating heart block	XLR	FSGS	[25]
* PAX2*	Paired box protein 2	Isolated SRNS (adult-onset)	AD	FSGS	[26]
*LMNA*	Lamin A and C	Familial partial lipodystrophy, FSGS	AD	FSGS	[27]
*WDR73*	WD repeat domain 73	Galloway-Mowat syndrome	AR	FSGS, DMS	[28]
Cytoskeletal, scaffold and membrane proteins					
*ACTN4*	α-actinin 4	SRNS (late onset)	AD	FSGS	[29]
*MYH9*	Myosin heavy chain 9, non-muscle	MYH9-related disorders, SRNS	AD	FSGS	[30]
*INF2*	Inverted formin 2	SRNS, Charcot-Marie-Tooth disease	AD	FSGS	[31]
*MYO1E*	Myosin 1E	SRNS	AR	FSGS	[32]
*MAGI2*	Membrane Associated Guanylate Kinase, inverted 2	CNS, SRNS	AR	MCD	[33]
*ANLN*	Anillin actin binding protein	SRNS (adult-onset)	AD	FSGS	[34]
*ARHGAP24*	Rho GTPase-activating protein 24	SRNS (adult-onset)	AD	FSGS	[35]
*ARHGDIA*	Rho GDP dissociation inhibitor alpha	SRNS (CNS), seizures, cortical blindness	AR	FSGS	[36]
*KANK 1/2/ 4*	Kidney ankyrin repeat-containing protein	SRNS +/− haematuria	AR	FSGS	[37]
*SYNPO*	Synaptopodin	FSGS	AD	FSGS	[38]
*PTPRO*	Protein-tyrosine phosphatase-R O	SRNS (childhood onset)	AR	FSGS, MCD	[39]
*EMP2*	Epithelial membrane protein 2	SRNS (childhood onset)	AR	FSGS	[40]
*APOL1*	Apolipoprotein L1	Susceptibility to SRNS	Biallelic	FSGS	[41]
*CUBN*	Cubilin	SRNS	AR	FSGS	[42]
*PODXL*	Podocalyxin	FSGS	AD	FSGS	[43]
Glomerular basement membrane-associated proteins					
*LAMB2*	Laminin subunit β2	Pierson syndrome, isolated SRNS	AR	DMS, FSGS	[44]
*ITGB4*	Integrin β4	Epidermolysis bullosa, SRNS, lung disease	AR	FSGS	[45]
*ITGA3*	Integrin α3	Epidermolysis bullosa, SRNS, lung disease	AR	FSGS	[46]
*COL4A3*/*4*/*5*	Type IV collagen α3, α4, α5	Alport syndrome	AD, AR, XL	FSGS	[47]
*GPC5*	Glypican 5	NS (adult onset)	Risk gene	Variable	[48]
*CD151*	CD151 antigen	FSGS, bullous skin lesions, deafness	AR	FSGS	[49]
Mitochondrial proteins					
*COQ2*	Coenzyme Q2	CoQ_10_ deficiency, SRNS +/− encephalopathy	AR	CG	[50]
*COQ6*	Coenzyme Q6	CoQ_10_ deficiency, SRNS and deafness	AR	FSGS, DMS	[51]
*PDSS2*	Prenyl-diphosphate synthase subunit 2	CoQ_10_ deficiency, SRNS, Leigh syndrome	AR	FSGS	[52]
*ADCK4*	AarF domain containing kinase 4	CoQ_10_ biosynthesis disruption	AR	FSGS	[53]
*MTTL1*	Mitochondrial tRNA 1	MELAS, diabetes, deafness, SRNS	Mitochondrial	FSGS	[54]
Lysosomal and endocytic proteins					
*SCARB2*	Scavenger receptor class B, member 2	Action myoclonus-renal failure syndrome	AR	FSGS	[55]
*OCRL1*	Oculocerebrorenal syndrome of Lowe	Dent-2 disease, Lowe syndrome, SRNS	XLR	FSGS	[56]
Metabolic and cytosolic proteins					
*ZMPSTE24*	Zinc metallopeptidase STE24	Mandibuloacral dysplasia	AR	FSGS	[57]
*PMM2*	Phosphomannomutase 2	Congenital defect of glycosylation	AR	CG	[58]
*ALG1*	Asparagine-linked glycosylation 1	Congenital defect of glycosylation	AR	FSGS	[59]
*TTC21B*	Tetratricopeptide repeat protein 21B	FSGS	AR	FSGS	[60]
*CFH*	Complement factor H	SRNS	AR	FSGS	[61]
*DGKE*	Diacylglycerol kinase epsilon	NS	AR	FSGS	[62]

AD, Autosomal dominant; AR, autosomal recessive; CG, collapsing glomerulopathy; CNS, congenital nephrotic syndrome; DMS, diffuse mesangial sclerosis; FSGS, focal segmental glomerulosclerosis; MCD, minimal change disease; MELAS, Mitochondrial encephalomyopathy, lactic acidosis, and stroke-like episodes; NS, nephrotic syndrome; PMS, progressive mesangial sclerosis; PTRD, proximal tubule radial dilatation; SRNS, steroid-resistant nephrotic syndrome; XL, X-linked

With the ever-increasing number of genes implicated in SRNS and significant variability in clinical phenotype, clinicians face difficulties when presented with a child with SRNS. Mutation detection in such patients allows for a more personalised treatment approach; that is, the possibility of avoiding immunosuppressive therapy, thereby preventing associated side effects, and the potential to better predict post-transplant reoccurrence. A genetic diagnosis may also allow screening for, and early management of, associated medical conditions, such as glaucoma in Nail–Patella syndrome. In addition, a molecular diagnosis offers scope for more accurate genetic counselling, risk stratification and prenatal diagnosis for affected families. Currently, there are no clear guidelines detailing the clinical utilisation, relevance and cost-effectiveness of mutational screening for children with SRNS. Here, we discuss the most common causes of monogenic SRNS and link these causes to clinical phenotypes. We discuss the indications for genetic testing and propose a clinically useful approach for mutational screening in SRNS, with particular reference to who should undergo genetic testing, when this should be performed and how this should be carried out.

## Podocyte biology

**Fig. 1:**
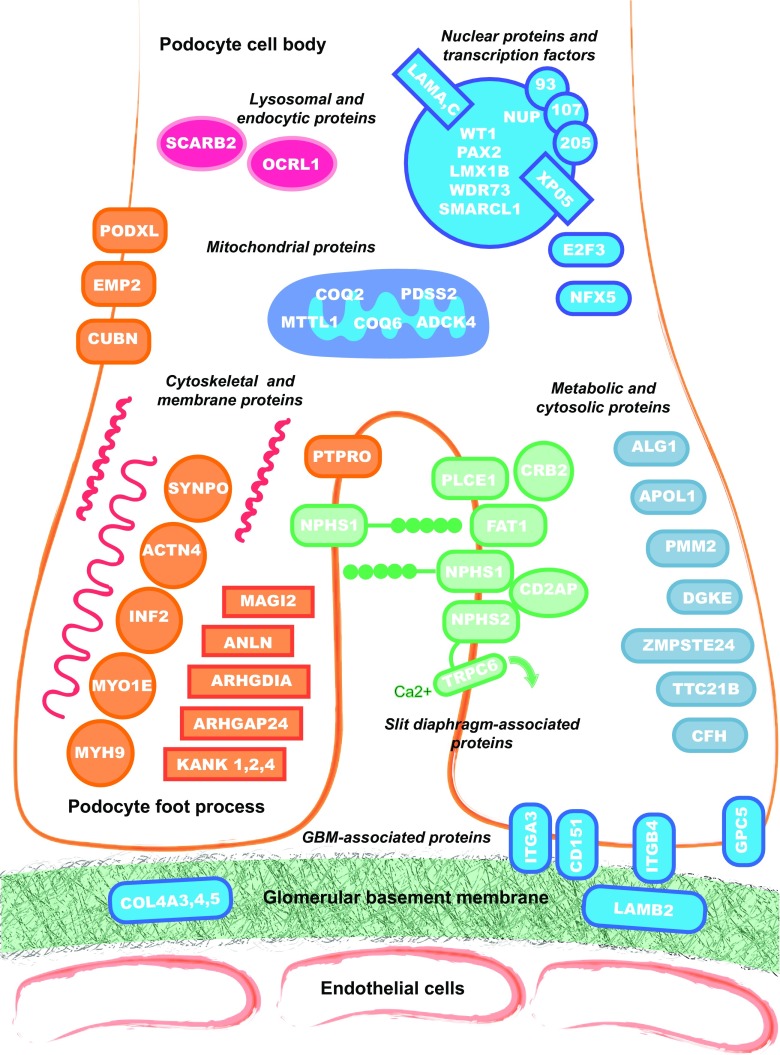
Genetic mutations associated with steroid-resistant nephrotic syndrome (SRNS) grouped according to location and function within the glomerular filtration barrier. For full names of proteins encoded by genes, please refer to Table 1

The GFB is composed of three interacting layers: the fenestrated endothelial cells, the glomerular basement membrane and the outer podocyte layer. Podocytes are highly specialised epithelial cells, and their interdigitating foot processes connect to form the slit diaphragm, a unique multi-protein cell junction structure which, through regulation of podocyte function, controls the ultrafiltration of molecules. Genetic advances in SRNS have unveiled dysfunction of podocyte- and slit diaphragm-associated proteins in the pathogenesis of proteinuria, highlighting their importance in maintaining GFB integrity. The discovery began with genes encoding the slit diaphragm proteins nephrin (*NPHS1*) and podocin (*NPHS2*) [11, 12]. Since then, linkage analysis and next generation sequencing (NGS) have permitted the identification of over 50 genes implicated in SRNS, and this number continues to increase. Interestingly, the majority of encoded proteins map to distinct structural protein complexes and signalling pathways within the podocyte (Fig. 1). A thorough functional analysis of these proteins is beyond the scope of this review, but readers are directed elsewhere for a more detailed evaluation [64].

## Genotype–phenotype correlations in monogenic SRNS

Monogenic SRNS can be inherited in an autosomal recessive, autosomal dominant or mitochondrial manner, and can occur as an isolated renal disease or as part of a multisystem disorder. Most cases of recessive disease are characterised by an early onset and high penetrance, and they are not infrequently associated with extra-renal malformations. In contrast, the main causes of dominant disease are associated with a later onset and incomplete penetrance, and the patients may remain asymptomatic. The genes associated with SRNS identified to date and their associated phenotypes are presented in Table 1. In the following sections, we outline the most common monogenic causes of SRNS, according to age of onset.

### Congenital NS

Congenital NS (CNS), which presents within the first 3 months of life, is commonly associated with causative mutations. Indeed, mutations have been identified in 75–100% of cases of CNS [8, 10, 65, 66]. Causative mutations appear to largely occur in one of five genes (*NPHS1*, *NPHS2*, *WT1*, *LAMB2* and *PLCE1*). *NPHS1*, encoding nephrin, is the main gene implicated in CNS, and mutation is responsible for the autosomal recessive Finnish type (CNF), which typically has a severe phenotype with massive proteinuria and rapid progression to ESRD [11]. However, the *NPHS1* mutation detection rate remains high amongst non-Finnish cases of CNS [8, 10, 65]. Mutations in the *NPHS2* gene, encoding podocin, are also responsible for a significant number of CNS cases, and the phenotype varies from the severe CNF presentation to milder disease with onset of proteinuria occurring later than in those with *NPHS1* mutations [4, 66, 67]. Mutations in the *PLCE1*, *WT1* and *LAMB2* genes have also been detected in patients presenting with isolated CNS; mutations in these genes will be discussed in more detail in the next section.

### Infantile and childhood NS

Monogenic NS, which presents in infancy (from 4 to 12 months of life) and during childhood, is most commonly attributed to mutations in the *NPHS2* gene, encoding podocin [8, 68]. There is a recognised genotype to phenotype correlation that explains this phenotypic variability [12, 66, 69]. Recent whole-exome sequencing performed on a paediatric cohort of SRNS patients revealed the mean age of onset associated with *NPHS2* mutations to be approximately 6 years [8]. That said, it is clearly important to consider *NPHS2* mutation as a cause for SRNS in a child of any age and, conversely, to expect a low rate of *NPHS2* mutations in certain ethnic groups, namely Chinese, Japanese and Korean [10, 70]. Additionally, *NPHS1* mutations have been identified in infants and children presenting with SRNS, with the rarer hypomorphic mutations being associated with a milder late-onset phenotype [8, 71, 72]. Thus, mutations in this gene should be considered in all paediatric age groups.

Mutations in *PLCE1* (encoding phospholipase C epsilon-1) typically cause isolated DMS, with patients presenting with severe, early-onset SRNS and rapid progression to ESRD [13]. Again, significant clinical heterogeneity exists, with *PLCE1* mutations manifesting from birth and throughout childhood, with both FSGS or DMS found histologically [73, 74].

*WT1* encodes Wilms’ tumour 1, a transcription factor and key kidney development gene. Mutations in *WT1* cause isolated and syndromic SRNS, which will be discussed later in this review. Previous studies have estimated *WT1* mutations to account for approximately 6% of sporadic SRNS cases in childhood, manifesting at any age, depending on the underlying mutation [8, 66, 75, 76]. Genotype–phenotype correlations have been drawn from specific *WT1* mutations. Of these, certain mutations cause early-onset, severe disease with DMS histologically, or alternatively, late-onset SRNS with FSGS histologically and slower progression to ESRD [75]. Interestingly, isolated SRNS may result from a wide range of *WT1* sequence variations, which are associated with variable expression and incomplete penetrance [75]. It is important to stress that *WT1-*related nephropathy may be an isolated disease with no associated comorbidities or it may be an initial symptom of syndromic SRNS with extra-renal features manifesting later.

Although mutations in *TRPC6* and *ACTN4* are typically associated with autosomal dominant late-onset disease, as discussed below, there are reports of both infantile and childhood-onset SRNS caused by mutations in these genes.

### Late-onset NS

Autosomal dominant SRNS typically presents later in life, in adolescence or adulthood, and has significant phenotypic variability. The overall mutation detection rate remains substantial, approaching 25% in adolescence and 12% in adulthood, but it is lower than cases presenting earlier in childhood [8, 65]. The main genes implicated in late-onset SRNS, which presents in adolescence, include *NPHS2*, *TRPC6*, *INF2* and *ACTN4* [31, 65, 68, 77, 78]. *TRPC6*, encodes a transient receptor potential cation channel and was originally identified as causing autosomal dominant FSGS, presenting in adolescence and early adulthood and showing relatively rapid progression to ESRD. Since then, *TRPC6* mutations have been implicated in childhood-onset FSGS, and even SRNS presenting within the first year of life, with variable disease severity [8, 10, 77, 79, 80]. Similarly, mutations in *ACTN4*, which encodes alpha-actinin 4, typically cause late-onset FSGS with slow progression to ESRD [29, 81], but mutations in this gene have been reported in children presenting with SRNS and rapid progression to ESRD [8, 82]. Mutations in *INF2*, encoding inverted formin 2, were originally identified in patients with autosomal dominant SRNS, with age of onset ranging from adolescence and throughout adulthood [31, 83]. Although *INF2* mutations typically result in isolated FSGS, they have also been detected in a subgroup of patients with associated Charcot–Marie–Tooth neuropathy [84]. Specific *NPHS2* mutations may manifest late, in adolescence or adulthood; the common variant R229Q may result in late-onset SRNS in compound heterozygotes with specific second mutations [85].

### Syndromic SRNS and mitochondrial disorders

Syndromic SRNS is associated with extra-renal manifestations and most commonly occurs due to mutations in genes encoding nuclear proteins (*WT1*, *LMX1B*, *SMARCL1*, *WDR73*), glomerular basement membrane and adhesion components (*LAMB2*, *ITGA3*, *ITGB4*), actin cytoskeleton components (*MYH9*) and lysosomal (*SCARB2*) and mitochondrial proteins (*COQ2*, *COQ6*, *PDSS2*, *MTTL1*, *ADCK4*). Table 2 lists the major extra-renal manifestations associated with gene defects causing syndromic SRNS; if extra-renal manifestations are present, it is highly likely that a causative mutation will be identified. A full description of syndromic SRNS is beyond the scope of this review but readers are directed to several detailed reviews for a thorough discussion [5, 20, 21, 44, 45, 50, 55, 84, 86, 87].

**Table 2:** Syndromic steroid-resistant nephrotic syndrome and associated extra-renal manifestations

Gene	Disease	Extra-renal manifestations
*WT1*	Denys–Drash syndrome	Urogenital abnormalities, ambiguous genitalia, nephroblastoma
	Frasier syndrome	Gonadoblastoma, male pseudohermaphroditism
*LAMB2*	Pierson’s syndrome	Ocular abnormalities; microcoria
*LMX1B*	Nail–Patella syndrome	Skeletal defects, hypoplastic nails, absent patella, glaucoma
*SMARCL1*	Schimke immune-osseous dysplasia	Spondyloepiphyseal dysplasia, T cell immunodeficiency, cerebral infarcts, skin pigmentation
*SCARB2*	Action myoclonus renal failure	Progressive myoclonic epilepsy, tremor, ataxia
*COQ2*	CoQ_10_ deficiency	Progressive encephalomyopathy
*COQ6*	CoQ_10_ deficiency	Sensorineural hearing loss
*PDSS2*	Leigh syndrome	Hypotonia, ataxia, deafness, growth retardation
*WDR73*	Galloway-Mowat syndrome	Microcephaly, psychomotor impairment, seizures, hypotonia
*MTTL1*	MELAS	Myopathy, encephalopathy, lactic acidosis, stroke-like episodes, diabetes, deafness
*ITGA3*	Epidermolysis-associated	Epidermolysis bullosa, interstitial lung disease
*ITGB4*	Epidermolysis-associated	Epidermolysis bullosa, pyloric atresia
*MYH9*	MYH9-related syndromes	Macrothrombocytopenia, mental retardation, sensorineural deafness, cataracts
*INF2*	Charcot–Marie–Tooth	Chronic peripheral motor and sensory neuropathy
*ZMPSTE24*	Manibuloacral dysplasia	Mandibular and clavicular hypoplasia, cutaneous atrophy, lipodystrophy, acro-oestolysis

For names of encoded proteins and associated histology, please consult Table 1

*WT1* mutations are associated with a spectrum of significant extra-renal manifestations, including urogenital abnormalities and malignancy. Mutations in the KTS (splice insertion) site are associated with Frasier syndrome, characterised by childhood-onset SRNS, histologically characterised FSGS, male-to-female sex reversal and increased risk of gonadoblastoma [18, 75]. Missense mutations in exons 8 and 9 (affecting the zinc finger domains) are associated with Denys–Drash syndrome, characterised by infantile-onset SRNS, histologically characterised DMS, sex reversal, gonadoblastoma and Wilms’ tumour [75]. Large genomic rearrangements disrupting *WT1* and the neighbouring *PAX6* result in WAGR syndrome (Wilms’ tumour, aniridia, genito-urinary abnormalities and mental retardation) [75]. Although these genotype–phenotype correlations have been clearly described, it is important to note that there remains significant phenotypic variability with respect to extra-renal manifestations of *WT1*-associated disease. Furthermore, histopathological heterogeneity is noted even amongst carriers of the same genetic abnormality [75].

*LAMB2* and *LMX1B* mutations typically cause Pierson syndrome and Nail–Patella syndrome, respectively, but have also been identified in isolated congenital, infantile and childhood-onset SRNS [88, 89], and mutation in these genes should therefore be considered to be causative in these age groups.

Isolated SRNS may also be seen, although rarely, in certain mitochondrial cytopathies, including MELAS (mitochondrial myopathy, encephalopathy, lactic acidosis and stroke-like episodes) caused by *MTTL1* mutations [54] and coenzyme Q_10_ deficiency [90]. Coenzyme Q_10_ deficiency due to mutations in *COQ2* and *COQ6* may cause an isolated or syndromic nephropathy [51]. Mutations in *PDSS2* cause Leigh syndrome but may also cause isolated SRNS [52].

*ADCK4* mutations have been found to cause isolated SRNS with histologically characterised FSGS, manifesting from infancy through to early adulthood [53]. In contrast to previous studies [9], in a large multicentre cohort of Chinese paediatric patients with SRNS, *ADCK4* was found to be the most commonly mutated causative gene, responsible for SRNS presenting as early as the congenital period, but most frequently during childhood [10].

Interestingly, there have been reports of patients with coenzyme Q_10_ deficiency and *ADCK4* mutation whereby interventional treatment with COQ_10_ supplementation has been shown to modify disease progression [53, 90].

## Genetic testing; why?

Genetic testing in NS has important clinical and non-clinical implications. Confirmation of a genetic defect can personalise SRNS management by means of predicting clinical course, weaning immunosuppression, avoiding renal biopsy, planning renal transplantation, providing genetic counselling and offering potential antenatal and pre-symptomatic diagnosis. In addition to these clinical benefits, identification of known and novel pathogenic variants implicated in monogenic SRNS will better define genotype–phenotype correlations, advance our understanding of the GFB and generate novel avenues for the *in vitro* and *in vivo* study of the pathophysiological mechanisms of proteinuria.

### Immunosuppression

Several studies highlight that monogenic SRNS is largely steroid resistant, irrespective of the causative mutation [6, 69, 91]. Subsequent management of such patients often includes various immunosuppressive agents, all with associated adverse side effects and often limited clinical benefit [6, 91]. Indeed, in a recent paediatric cohort of sporadic SRNS, none of those with an identified causative mutation responded to immunosuppressive agents, compared to almost 60% of those without a mutation [6]. However, there are reports in the literature of a partial response to steroids, ciclosporin therapy or calcineurin inhibitors in patients with specific *WT1*, *NPHS2*, *PLCE1* and *TRPC6* mutations or mutations in the regulators of Rho-like GTPase (*ARHGDIA*, *KANK 1*, *KANK 2* and *KANK 3*) [13, 92–94]. Nonetheless, given that responsiveness to immunosuppression is unlikely in monogenic SRNS, upon receiving a positive genetic test result, clinicians should consider weaning immunosuppression if no clinical benefit has been demonstrated, thus protecting patients from the potentially devastating side effects of such treatments. It is important to note that there have been reports of patients with monogenic SRNS partially responding to secondary immunosuppression [13, 92–94]; in such cases, it would be important to continue therapy whilst clinical improvement continues.

### Novel interventional therapy and monitoring

The discovery of rare monogenic causes of SRNS have revealed a small but significant cohort whose disease may be amenable to specific interventional treatment, thereby avoiding lengthy immunosuppression and delaying progression to ESRD. Patients with disease-causing mutations in genes encoding enzymes of the coenzyme Q_10_ pathway (*COQ2*, *COQ6* and *ADCK4*) and in the *CUBN* gene may respond to treatment with coenzyme Q_10_ and vitamin B12, respectively. Likewise, patients with *ARHGDIA* mutations, through modulation of Rac I–mineralocorticoid interactions, could theoretically respond to eplenerone (a mineralocorticoid-receptor antagonist) [36].

As highlighted in Table 2, many syndromic forms of SRNS have associated medical problems that may benefit from early recognition and management. An example is the *WT1* mutation, which can predispose to malignancy, and the detection of such mutations should trigger monitoring for associated Wilms’ tumour and gonadoblastoma. Given the latter entity is largely associated with sex reversal, a karyotype analysis should also be performed, especially in phenotypically female patients presenting with SRNS and primary amenorrhoea.

### Renal biopsy

Renal histology has historically been utilised as a key diagnostic and prognostic criterion for children with SRNS, but emerging evidence reveals significant histological heterogeneity amongst monogenic causes of SRNS, demonstrating that biopsy findings may not correlate with genetic results [8, 95]. Furthermore, there does not appear to be a notable difference in the frequency of histological lesions found in patients with or without a recognised genetic cause [6]. For these reasons, in cases of primary SRNS, rapid genetic testing has the potential to obviate the need for renal biopsy for diagnostic purposes and serves as a less invasive diagnostic method; this is of particular significance in the younger SRNS cohort, for whom a genetic aetiology is more likely. In the event of rapid genetic testing being inaccessible, renal histology may direct clinicians towards the most likely “culprit” gene; for example, if DMS is detected in an infant presenting with SRNS, it would be prudent to perform mutational analysis on certain genes (*LAMB2*, *WT1*, *NPHS1*, *PLCE1*) preferentially over others. However, clinical indications for renal biopsy do remain, such as atypical features suggestive of lupus nephritis, with histology providing useful information. Additionally, a histological diagnosis enables phenotype patterns to become better established and is therefore useful from a clinical research perspective.

### Disease reoccurrence post-transplantation

There is a high risk of progression to ESRD in monogenic SRNS, with many patients requiring renal transplantation [96]. Several studies have highlighted a low risk of disease reoccurrence post-transplant when a genetic aetiology has been confirmed [6, 8, 68, 69, 96, 97]. Conversely, there is a high risk of post-transplant disease reoccurrence in the idiopathic group; this is postulated to be caused by circulating factors [98, 99]. Given that the likelihood of post-transplant reoccurrence is minimal for genetic SRNS; after excluding the mutation in parents, a parental transplant can be planned, although in our experience this typically follows bilateral nephrectomy and an interval of time on dialysis. Post-transplantation prognosis is improved with living donor transplantation, which is associated with prolonged graft survival and decreased rejection rates as compared to deceased donor kidney transplantation.

### Genetic counselling

Genetic counselling prior to testing should ensure that families are informed regarding potential outcomes and limitations of the chosen genetic test, including the discovery of variants of unknown significance and the potential for incidental findings. Possible benefits, including changes to medical management, should be discussed, as well as potential harms, including privacy, legal and social implication. These subjects are covered in the next section.

Making a molecular diagnosis has important implications for a family. It may enable accurate discussion of recurrence risk in future children and potential identification of pre-symptomatic individuals at risk [4]. Early referral to a clinical genetics service can facilitate identification of individuals at risk and genetic testing of family members as well as counselling in terms of family planning, prenatal diagnosis and pre-implantation genetic diagnosis. Genetic screening in unaffected family members may be additionally important when planning a living related donor (LRD) renal transplant, especially in the case of autosomal dominant disease. When inheritance of a severe disease-causing mutation is likely, pre-symptomatic testing for proteinuria and genotyping at birth are avenues that should be discussed and offered to affected families. Ethical considerations of causing potentially unnecessary anxiety should be addressed, and families should be counselled on the benefits and risks of such an approach; that is, the benefits of providing a timely genetic diagnosis, and therefore active clinical management, versus the risks of testing for a disease which may never manifest or be mild in the event of incomplete penetrance or variable expression. Prenatal diagnosis could be offered in families with a known risk of severe NS, such as CNS, or in cases where elevated alpha-fetoprotein levels have been detected in maternal serum or amniotic fluid. It can be used to allow the family to make an informed decision about continuing a pregnancy or to allow preparation both by the family and medical professionals for the birth of an affected child.

### Risks of genetic testing

In addition to the ethical and emotional considerations that must be addressed during genetic counselling, there are several other risks to the patient and their family who are considering genetic testing or receiving a genetic diagnosis. In the UK, genetic testing is usually paid for by the National Health Service, and in countries with a privatised medical system, health insurance policies often cover the cost of genetic testing performed at the request of a doctor. However, this is not always the case, and in some situations the significant cost, which can be over US$2000, must be covered by the family. Furthermore, upon receiving a genetic diagnosis, the fear of insurance discrimination and the associated costs of enhanced insurance premiums represent a significant emotional and financial burden. Although there is legislation in place which protects those with a genetic disease from discrimination by health insurers, this does not always extend to protect patients from employment discrimination or the amplified costs of life, disability and long-term care insurance [100]. As part of the genetic counselling process, these issues should be discussed with affected families and informed consent obtained prior to genetic testing. A thorough discussion regarding the barriers to genetic testing in public health is beyond the scope of this review, but readers are directed to several useful articles for further information [100–104].

## Genetic testing; who?

Having discussed the benefits of identifying a causative mutation in patients with SRNS, it is important to note that the overall burden of monogenic SRNS has yet to be fully delineated. Recent evidence estimates that a genetic aetiology is detected in approximately 30% of cases. A negative result does not exclude genetic disease as mutations may be missed, with sensitivity for genes covered by the test depending on methodology and analysis used. Alternatively, a mutation may be present in a gene not covered by the chosen test, for example a novel genetic association. This, combined with the profound clinical and pathological heterogeneity of genetic and idiopathic SRNS, highlights that universal genetic testing in SRNS is inappropriate and unlikely to be cost-effective. Rather, mutational screening should be directed towards those in whom a genetic aetiology is likely and should therefore be reserved for patients presenting with primary SRNS.

### Indications for genetic testing

When accessible and affordable, mutational screening should be performed in all children presenting with primary SRNS. Even in young adults, the likelihood of detecting a causative mutation remains substantial, and when cost allows, mutational screening should also be offered to this cohort. However, when such an inclusive approach is not possible, there are certain indications in which a genetic cause for SRNS becomes more likely, and mutational screening should be performed as a priority. Given that the likelihood of detecting a causative mutation is inversely related to age of disease onset [105], mutational screening becomes increasingly important the earlier the disease manifests. Genotype–phenotype correlations clearly demonstrate that mutations in recessive genes are more frequently implicated in early-onset disease and that mutations in dominant genes are more frequently implicated in adult-onset disease. Although there may not be an obvious family history in early-onset disease, a positive family history in any age group indicates that monogenic SRNS is likely and should trigger mutational screening. Additionally, the likelihood of finding causative recessive mutations correlates directly with the degree of consanguinity [80]; thus, a history of consanguinity should prompt mutational screening. Finally, the presence of extra-renal manifestations suggestive of an underlying genetic syndrome (Table 2) makes screening of associated genes advisable.

The clinical indications for genetic testing in SRNS can be summarized as follows:

- Congenital or infantile-onset NS
- Childhood-onset NS
- Family history of NS
- Consanguinity
- Extra-renal manifestations.

## Genetic testing; when?

Before undertaking genetic testing for SRNS, it is important that the potential detection of a causative mutation is likely to aid in diagnosis, alter clinical management, inform likely prognosis and provide information when stratifying risk for family members and delivering genetic counselling. In congenital- and infantile-onset NS, genetic testing should be considered before commencing immunosuppressive therapy or performing renal biopsy. Similarly, when genetic testing can be performed in a timely manner, early confirmation of a genetic diagnosis in childhood-onset SRNS would minimise the adverse effects of current therapies on the growing child. Pre-transplantation genetic testing will provide clinicians with information that may be helpful in predicting the risk of post-transplant reoccurrence and will therefore guide pre- and post-transplant management, especially when considering LRD kidney transplantation from family members.

To summarise, we suggest that genetic testing should be considered when important clinical decisions need to be made regarding the need for renal biopsy, the intensity and duration of immunosuppression and pre-transplantation therapy, and when syndromic SRNS is suspected.

## Genetic testing; how?

Traditionally, genetic testing in diagnostic laboratories has employed Sanger sequencing, frequently in association with exon copy number analysis, to assess specific disease-related genes individually. In genetically heterogeneous disorders, with multiple causal genes, such as SRNS, this method can be expensive and time-consuming owing to the cost of screening multiple individual genes. The advent of high-throughput massively parallel sequencing (NGS methods) allows for a higher diagnostic yield, time savings and a reduction in cost [105, 106]. Typically, diagnostic laboratories utilise a targeted capture of a ‘panel’ of genes of interest followed by sequencing on an NGS platform. Sanger sequencing still plays an important role for the confirmation of genetic variants identified via NGS and filling in of regions of poor coverage. The limitations of Sanger sequencing include the need to ensure both adequate coverage of regions of interest and adequate analysis to detect copy number variants such as exonic deletions. As with most Sanger sequencing approaches, this method will miss deep intronic or regulatory region variants unless specifically targeted.

Whole-exome sequencing (WES) or whole-genome sequencing (WGS) employ NGS methods to attempt to sequence the coding portion of the genome (the exome) or the entire genome, respectively. This approach is not limited to known candidate genes and therefore has the ability to identify mutations in novel genes, thereby expanding the heterogeneity of SRNS and enhancing our understanding of the pathogenesis and molecular mechanisms of proteinuria. WES is increasingly being implemented in the clinical setting, but its widespread application is limited by the amounts of data generated and the requirements for robust bioinformatics support and assessments of the pathogenicity of larger numbers of variants. When targeted capture utilised for WES gives sufficient coverage of the ‘Mendeliome’, an *in silico* panel of genes can be analysed to give similar results to a targeted capture approach, whilst giving the flexibility to ‘open’ the data if the initial analysis does not find a variant of interest, or in light of novel genetic associations. A similar approach can be utilised with WGS, with the potential advantage of improved coverage, coverage of regulatory and intronic regions and improved analysis of copy number and structural rearrangements, but with the disadvantages of increased cost and substantially increased data and variant volume.

WES and WGS are hampered by the fact that large numbers of genetic variants are identified, including variants of unknown significance and incidental or secondary findings. These findings raise a number of ethical and practical issues relating to consent, data storage and analysis, all too extensive to cover here. The American College of Medical Genetics and Genomics has published guidance on reporting secondary findings.

When compared to WES or WGS, the cost-effectiveness of NGS using a targeted gene panel analysis has greater clinical application in SRNS, as it produces a more feasible dataset for bioinformatics analysis which is functionally interpretable in a clinical setting.

## Application to SRNS

Currently, clinical phenotyping combined with targeted NGS panel analysis is the most cost-effective and clinically useful approach for mutational screening in SRNS. This method enables clinicians to quantify and stratify likely response to immunosuppression, rate of progression to ESRD and risk of post-transplant reoccurrence. Using NGS technology, most monogenic SRNS genes (approximately 40–50 genes per panel) can be analysed within 6 weeks and at a competitive price compared to Sanger methods [6, 107]. There are several commercial indication-driven SRNS gene panels currently in use around the world, with many laboratories conducting entire or targeted sequence analysis, antenatal testing and carrier screening for SRNS genes. Indeed, an internet search (www.genetests.org) reveals at least 12 laboratories worldwide offering extended NGS panels for SRNS with an average turnaround of 3–6 weeks and associated cost ranging from $1000 to $2200. Comparatively, Sanger sequencing for individual genes or small panels of genes (approximately 5 genes) has a slightly quicker turnaround of 2–4 weeks and, depending on the size of the gene, costs $450–$1000 per individual gene.

In certain circumstances where NGS technology is inaccessible or unaffordable, and a disease-causing mutation is highly likely in a specific gene, as suggested by the presence of extra-renal manifestations or a positive family history, Sanger sequencing methods remain an important diagnostic tool. It is important to stress that employing genotype–phenotype correlations alone to direct mutational screening using Sanger methods is only cost-effective, and clinically beneficial, provided a causative mutation is identified early in the screening process.

## Approach to mutational screening

**Fig. 2:**
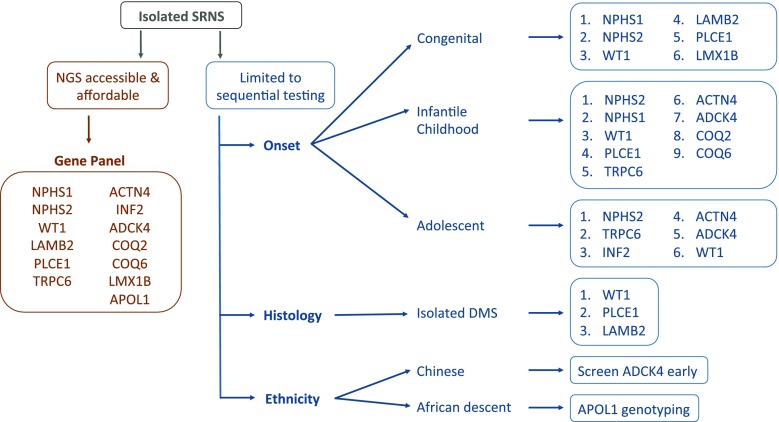
Mutational screening in children with isolated SRNS. If next-generation sequencing (NGS) technology is accessible, screening should utilise a gene panel including, but not limited to, the most common monogenic causes of SRNS. If NGS technology is inaccessible, genes should be screened in numerical order of frequency per age group. Ethnicity and histological findings should trigger preferential screening of certain genes. DMS Diffuse mesangial sclerosis. For names of genes and associated encoded proteins, please refer to Table 1

Our recommended approach to mutational screening in paediatric SRNS is demonstrated in Fig. 2. If NGS techniques are accessible and affordable, an extended SRNS gene panel including, but not limited to, the most common monogenic causes of SRNS for each age group should be screened (Fig. 2). For SRNS presenting in the congenital period, the panel should include the five most likely causative genes (*NPHS1*, *NPHS2*, *WT1*, *LAMB2* and *PLCE1*), and for those presenting in infancy or childhood, the gene panel should also include *TRPC6*, *ACTN4* and *ADCK4*. Similarly, for those presenting in adolescence, an extended gene panel would maximise the likelihood of identifying a causative mutation, and the panel should include the genes already mentioned, as well as *INF2*.

Patients with disease-causing mutations in genes encoding enzymes of the coenzyme Q_10_ pathway (*COQ2*, *COQ6* and *ADCK4*) may be amenable to treatment, and although these represent a rare group of patients, they are important to recognise and should therefore be included in the genetic screening panel for isolated SRNS presenting in any paediatric age group, including the first year of life. This is especially true for patients of Chinese, Japanese and Korean origin, as there appears to be an increased frequency of the *ADCK4* mutation in these populations [10, 70]. Additionally, given that the *APOL1* genotype represents a vulnerable population who present with more advanced disease [108], defining a patient’s *APOL1* genotype has important clinical implications, and mutational screening of this gene should be included in the gene panel, especially for patients of African descent.

If NGS technology is neither accessible nor affordable and clinicians are limited to sequential testing, genes should be screened in numerical order, for each age group, as depicted in Fig. 2. If renal histology is available and reveals isolated DMS, we suggest preferentially screening for mutations in *WT1*, *PLCE1* and *LAMB2*. In the presence of extra-renal manifestations, it is highly likely that a causative mutation will be detected, and genes should be selected for screening depending on the specific phenotype identified (Table 2). In view of the significant complications associated with *WT1* mutations, establishing a genetic diagnosis appears to be particularly important; it is therefore advisable to routinely screen *WT1* for mutations in isolated SRNS.

With the advent of NGS methods targeting large panels of genes, novel pathogenic variants in genes that are more rarely implicated in paediatric SRNS are being identified. Most recently, mutations in *SMARCAL1*, *CUBN*, *LMX1B*, *PODXL* and the nucleoporin genes, *NUP93* and *NUP107*, have been found to be causative of isolated SRNS presenting from infancy and throughout childhood [8, 10]. Moreover, mutations in genes which are usually linked to another renal phenotype (*DGKE*, *OCRL* and *COL4A3*) have been found to be causative of isolated SRNS presenting in childhood [8]. These findings highlight the importance of employing NGS technology in the diagnosis of monogenic SRNS through expanding current knowledge in the pathogenesis and molecular basis of proteinuria. For this reason, extended gene panels and if appropriate resources are available, WES/WGS techniques, should be considered wherever possible.

## Conclusion

Mutations of podocyte-associated genes account for approximately 30% of paediatric cases of SRNS. The younger the child at presentation, the higher the genetic diagnostic rate. Advances in genomic sequencing have improved our understanding of the molecular basis of NS and of the genetic heterogeneity of SRNS. A genetic diagnosis allows a “personalised” approach when investigating and managing patients and their families. That is, clinicians are more able to make decisions about weaning immunosuppression, avoiding renal biopsy and planning renal transplantation and geneticists can offer more informed genetic counselling. In a small but significant sub-group of patients with specific mutations, a genetic diagnosis may open avenues for interventional disease-modifying therapy and allow clinicians to detect and treat asymptomatic extra-renal manifestations early.

The clinical utility of such advances has been hindered by a lack of clear guidelines pertaining to mutational screening in SRNS. There exists profound clinical and pathological heterogeneity in monogenic SRNS, with mutations in the same gene and even identical mutations resulting in significant phenotypic variability. This heterogeneity renders sequential mutational screening using traditional Sanger sequencing a timely and costly process. Technological advances in genomic sequencing have led to the development of commercial, indication-driven gene panels which simultaneously sequence over 40 known SRNS-related genes. NGS panels are available worldwide, and with a similar turnaround time and slightly increased cost to traditional methods, they currently represent the most time- and cost-effective approach to mutational screening in SRNS. We propose that all children presenting with primary SRNS be screened for monogenic causative mutations using an extended gene panel; especially in cases of early-onset disease and those with a positive family history or history of consanguinity. If such an inclusive approach is not possible, we provide recommendations for sequential gene testing which direct the clinician towards the most frequently occurring causative mutation per age group, depending on available histology, presence of extra-renal manifestations and ethnicity.

NGS methods targeting large panels of genes, the whole exome or genome have allowed the identification of novel pathogenic variants and also novel genetic associations implicated in paediatric SRNS. This in turn enables *in vitro* and *in viv*o study of podocyte-associated proteins, further unravelling the pathogenic pathways of SRNS and providing important therapeutic targets to guide advanced medical management on a gene-specific basis.

## Key summary points

- Mutations of podocyte-associated proteins account for approximately 30% of SRNS in childhood.
- The likelihood of detecting a causative mutation is inversely related to age of disease onset.
- Monogenic SRNS displays significant phenotypic heterogeneity in terms of associated renal histology and clinical presentation.
- A definitive molecular diagnosis has important clinical implications, allowing for a personalised treatment approach.
- Recent advances in high-throughput sequencing have revolutionised genetic testing, and indication-driven gene panel analysis currently represents the most cost-effective approach for mutational screening in SRNS.
- Identification of novel SRNS genes and causative mutations will further unravel the pathogenic pathways of SRNS.
